# Anal high-grade squamous intraepithelial lesions: identification, classification, and management

**DOI:** 10.1093/bjs/znag006

**Published:** 2026-03-30

**Authors:** Emanuel Cavazzoni, Mariangela Desantis, Carlo Vivaldi, Hartmut Schäfer, Massimiliano Mistrangelo

**Affiliations:** School of Medicine, University of Perugia, Perugia, Italy; Policlinique Santa Maria, Groupe Kantys, Nice, France; Faculty of Medicine, University of Cologne, Cologne, Germany; Faculty of Medicine, University of Cologne, Cologne, Germany; Città Della Salute e Della Scienza, Università Degli Studi di Torino, Torino, Italy

## Abstract

Squamous intraepithelial lesions are premalignant conditions for anal cancer, the incidence of which is slowly increasing worldwide. These premalignant lesions are commonly found in high-risk individuals such as those who are immunosuppressed, and men who have sex with men. The development of squamous intraepithelial lesions is driven by chronic human papillomavirus infection that induces cellular proliferation and dysplastic modification in mucosal and cutaneous epithelia. Among a wide family of human papillomavirus strains, high-risk genotypes such as human papillomavirus 16 and 18 are responsible for most high-grade precancerous lesions and their subsequent progression to invasive cancer.

Historically, premalignant lesions have been described by a variety of different classification systems that has changed as our understanding of their pathophysiology has evolved. Precancerous lesions are currently classified based on the degree of dysplasia in the anal epithelium. Anal intraepithelial neoplasia I or low-grade squamous intraepithelial neoplasia (LSIL) describes a low-grade dysplastic lesion capable of spontaneous regression, while anal intraepithelial neoplasia III or high-grade squamous intraepithelial neoplasia (HSIL) describes those lesions with capacity for infiltration and malignant change. The need for active screening in high-risk groups is now established, as proactive treatment of HSIL significantly reduces the development of anal cancer.

High-resolution anoscopy is a diagnostic modality that allows for the identification and treatment of anal dysplasia, halting its progression to squamous cell carcinoma. Using vital dyes and dedicated instruments offering high magnification and resolution, it is possible to visualize and treat very small and/or flat premalignant lesions that would be invisible on standard diagnostic anoscopy. The evolution of artificial intelligence is set to further enhance its diagnostic ability, and shorten the learning curve for its implementation.

## Introduction

**Fig. 1:**
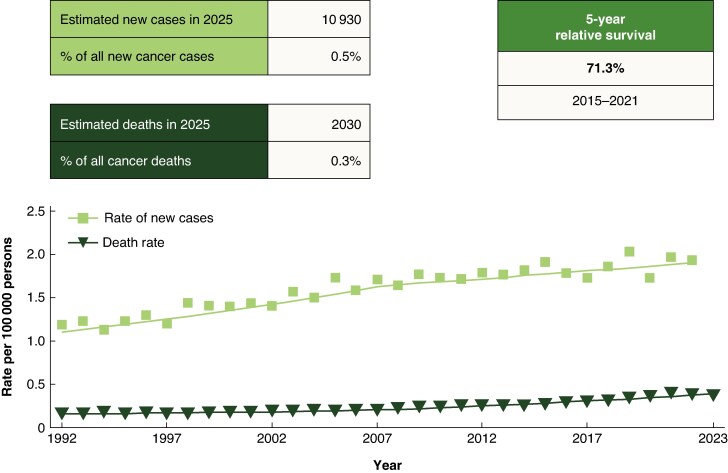
Incidence of anal cancer, US

Although considered rare, anal cancer has shown a progressive increase in incidence over recent decades^1^, with a frequency of more than two cases per 100 000 people and a 5-year mortality rate close to 30% (*Fig. 1*)^2^. The main reason for this is the spread of human papillomavirus (HPV) in the general population, partly due to changes in sexual habits, but also the growing population of immunosuppressed patients (not exclusively those with human immunodeficiency virus (HIV) infection) and their increasing life expectancy.

It is now well known that anal squamous cell carcinoma (ASCC) is preceded by premalignant lesions induced by chronic HPV infection in the anal canal and perianal region. These dysplastic lesions are known as squamous intraepithelial lesions (SILs) and tend to progress to invasive cancer in a highly variable percentage and typically over a long time. The natural history of anal cancer almost entirely mirrors that of cervical cancer, which notoriously takes decades to progress from HPV infection to high-grade dysplasia and finally to invasive cancer^3^. The HPV vaccination campaign will radically change the epidemiology of anal cancer. Based on statistical data regarding the reduction in the incidence of HPV infection in school-age vaccinated individuals before viral exposure, a significant reduction in all HPV-induced squamous cell carcinomas can be expected over the next 20–30 years^4,5^. During this time, it is crucial to prevent the development of ASCC in those individuals at higher risk, who unfortunately did not benefit from the vaccine’s prophylactic effect.

Fortunately, knowledge of the natural history of ASCC has grown such that this neoplasm could now be considered almost entirely preventable, given that patients at the greatest risk of developing squamous tumours are much more clearly identified and screening programmes to detect premalignant lesions at an early stage are increasing worldwide. Finally, there has been significant progress in the use and quality of high-resolution anoscopy (HRA), the diagnostic method that allows the visualization of anal dysplastic areas and, in many cases, their treatment with dedicated ablative instruments.

## Classification of anal premalignant lesions

A high-grade squamous intraepithelial lesion (HSIL) is an HPV-related squamous epithelial dysplasia and a precursor to invasive squamous cell carcinoma in multiple anatomic sites, including the anogenital tract.

The Lower Anogenital Squamous Terminology (LAST) Project for HPV-associated lesions provides guidelines for a unified nomenclature^6^. A two-tiered system, designating lesions as low-grade squamous intraepithelial lesions (LSILs) or HSILs, is recommended for reporting and describing the severity of dysplastic alterations that afflict the anal epithelium. In the anal canal, an LSIL correlates with anal intraepithelial neoplasia (AIN) previously described as AIN1 or anal condyloma, while an HSIL correlates with the previous definition of AIN2 and AIN3. The two-tiered system, based on consensus evaluation, more accurately reflects the biology of these HPV-related lesions. The use of immunohistochemistry better permits an adequate classification of AIN2 lesions: AIN2 p16-negative lesions are considered LSILs, while AIN2 p16-positive lesions are considered HSILs (*Table 1*).

**Table 1:** Current classifications of anal canal precancerous lesions

	LSILs	HSILs
Classification I	AIN1	AIN2–3
Classification II	AIN1 + AIN2 p16-negative	AIN2 p16-positive + AIN3

LSILs, low-grade squamous intraepithelial lesions; HSILs, high-grade squamous intraepithelial lesions; AIN, anal intraepithelial neoplasia.

An HSIL is the premalignant condition, principally arising in the transition zone of the anal canal or in the anal margin, and can progress to ASCC if left untreated, while an LSIL is now considered a dysplastic modification with a natural tendency for spontaneous regression and self-clearance, in a time frame and with a biological mechanism that are unfortunately not completely clear.

## Histopathology of HSILs

Cytology and HPV-DNA tests for anal premalignant lesions and cancer are recommended by the International Anal Neoplasia Society (IANS) to screen high-risk patients. As there is no specific reporting system for anal cytology, the Bethesda System for Reporting Cervical Cytology is currently used for anal dysplasia. The cytological classification of an anal Papanicolaou (Pap) smear is the same as that used for a cervical Pap smear, and includes negative for an intraepithelial lesion or malignancy, atypical squamous cells of undetermined significance (ASC-US), an LSIL, atypical squamous cells for which an HSIL cannot be ruled out (ASC-H), an HSIL, and invasive cancer. Unfortunately, the significance of LSIL, ASC-US, and HSIL diagnoses in anal cytology lies in the prediction of a so-called intraepithelial lesion, while the result of further biopsy (low-grade or high-grade lesion) cannot be reliably predicted^7–9^.

Clarke *et al*.^10^ performed a systematic review and meta-analysis of cytology and HPV-related biomarkers for anal cancer screening among different risk groups. The study revealed that the sensitivity and specificity of cytology and HPV testing were 81% and 62%, and 92% and 42% respectively. For cytology and HPV co-testing, the sensitivity and specificity were 93% and 33% respectively. Furthermore, limited data on other biomarkers (HPV E6/E7 mRNA and p16/Ki-67 dual stain) suggested higher specificity, but lower sensitivity compared with anal cytology and HPV.

LSILs mostly comprise condylomas and are characterized histologically by koilocytes and large cells with a cytoplasmic halo surrounding the nuclei that are the hallmark of HPV infection. HSIL cells are smaller with higher nuclear : cytoplasmic ratios. The main feature of an HSIL is the extension of dysplasia from the basal third of the epithelium to the upper two thirds. The presence of strong p16 immunostaining involving the entire epithelium is also characteristic of an HSIL. Other immunohistochemistry to distinguish an LSIL from an HSIL includes Ki-67, ProEx C, and CK7.

**Fig. 2:**
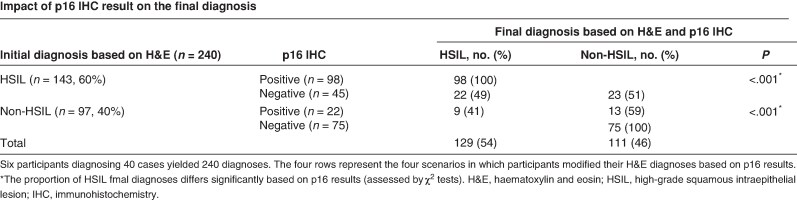
Role of p16 immunohistochemistry in the diagnosis of squamous intraepithelial lesions H&E, haematoxylin and eosin.

The LAST Project also underlined the role of biomarkers to better classify premalignant lesions, concluding that positive p16 immunohistochemistry (which reveals E6/E7-driven cell proliferation) supports the categorization of premalignant disease. Albuquerque *et al*.^11^ confirmed that the use of p16 immunohistochemistry in the analysis of AIN2 cases permitted downgrading of a substantial number of lesions (one-third in their series). Recently, Liu *et al*.^12^ confirmed that the use of p16 immunohistochemistry increased inter-observer agreement and, moreover, that negative expression of p16 can reduce unwarranted diagnoses of HSILs and therefore unnecessary treatment (*Fig. 2*). Finally, p16 immunohistochemistry is recommended as an adjunct to morphological assessment of biopsy specimens that are at risk of missed high-grade disease, defined as a prior cytological interpretation of an HSIL, ASC-H, ASC-US/HPV-16 positivity, or atypical glandular cells (not otherwise specified)^5^. The LAST Project classification also reports a new category of lesion between an HSIL and invasive squamous cancer, referred to as superficially invasive squamous cell carcinoma (SISCCA), which is characterized by an invasive depth of ≤3 mm from the basement membrane and a horizontal spread of ≤7 mm in maximal extent.

## Incidence, natural history, and risk factors

The incidence of anal premalignant lesions is difficult to estimate, but is heavily skewed by pre-existing conditions, particularly in high-risk populations. Both LSILs and HSILs have a clear association with HPV and are more prevalent in HIV-positive patients, men who have sex with men (MSM), solid organ transplant recipients, patients undergoing chronic immunosuppressive therapies, and women with a previous history of genital HPV-induced cancerous or premalignant lesions.

Despite a lack of data for the general population, a study of HIV-positive MSM screened with HRA for anal dysplasia showed that 40% had anal dysplastic lesions at the time of second examination within a time frame of 2–4 years^13^. In fact, rates of anal cancer in this population are dramatically elevated and ASCC can be found more commonly than those neoplasms which normally better correlate with corresponding age groups and sex. Finally, in recent years, there has been a trend towards diagnosis at a younger age, particularly among MSM, irrespective of their HIV status^14^.

## HPV-induced carcinogenesis

**Fig. 3:**
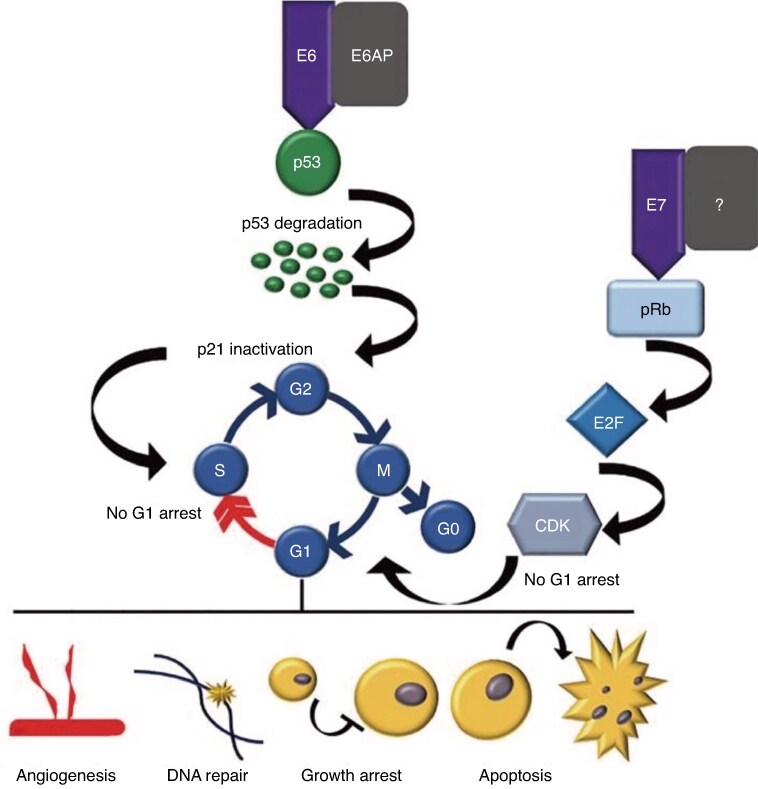
Mechanism of HPV-induced carcinogenesis Effect of HPV E6 and E7 viral oncoproteins on cellular processes. E6 and E7 viral oncoproteins can affect angiogenesis, DNA repair, growth arrest, and apoptosis by impairing G1 arrest. E6 binds to E6AP and subsequently binds to p53 and promotes its degradation by ubiquitination. Thus, the function of p53 as a tumour suppressor is diminished and the cell cycle is not properly controlled by p21, a CDK inhibitor that monitors the G1/S checkpoint. E7 leads to degradation of pRb, the retinoblastoma protein that functions as a tumour suppressor. Because of pRb inhibition by E7, cells can enter S phase without G1 arrest. HPV, human papillomavirus; CDK, cyclin-dependent kinase.

**Fig. 4:**
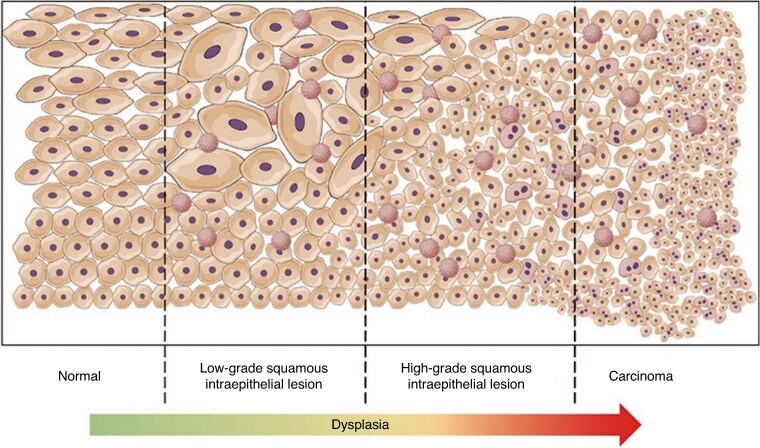
Chronic HPV infection and the progression to higher degrees of dysplasia Schematic of the progression of anal dysplasia. Starting on the left, normal epithelium is displayed. Further right, HPV-induced changes, including koilocytes, occur increasingly and atypical keratinocytes can be found in the upper layers of the epithelium. HPV, human papillomavirus.

SILs and 89–100% of anal cancers are caused by persistent infections with HPV. HPV infects basal epithelial cells within the transformation zone. The virus invades the cells by integrating its DNA with the normal cellular genome and disrupts their function by viral protein expression, in particular E6 and E7, which can act as oncoproteins (*Fig. 3*)^15^. These proteins interfere with the normal cell cycle pathway, leading to uncontrolled cell replication, which produces a defect in programmed cell death. HPV-infected cells can be recognized by the normal immune system, with subsequent clearing of the infection in most cases. A possible clearance interval of 150 days has been reported in several studies^16–18^. With persistent infection, particularly in the presence of a compromised immune system, there is potential for gene dysregulation resulting in increased cell growth and progression to a higher degree of dysplasia and possibly invasive carcinoma (*Fig. 4*)^19^.

## Screening and guidelines

Since the publication of a large American study involving 4446 patients, it is now clear that the treatment of people living with HIV (PLWH) who have histologically confirmed HSIL can significantly reduce the incidence of anal cancer^20^, definitively demonstrating that the adoption of screening programmes in high-risk populations can significantly reduce the incidence of ASCC^21^.

The IANS classifies individuals into risk groups A and B. Category A includes PLWH, particularly MSM and transgender women living with HIV aged ≥35 years, as well as all other PLWH aged ≥45 years. It also includes HIV-negative MSM and transgender women aged ≥45 years, individuals who received a solid organ transplantation (>10 years), and women with previous vulvar dysplasia or carcinoma. Category B includes women with cervical or vaginal intraepithelial neoplasia (CIN or VIN respectively) or carcinoma, individuals with anogenital warts, women with persistent cervical HPV-16 infection, and those undergoing immunosuppressive therapy^22^.

The recently published German and Austrian guideline on the screening of anal dysplasia in PLWH differs slightly from the IANS guidelines and includes women with CIN3 or VIN3, or men with penile intraephithelial neoplasia-grade 3, as well as PLWH with a CD4 nadir <200 cells/μl, within the equivalent of category A^19^. In general, all currently available guidelines reiterate that it is essential to monitor patients at high risk, especially if they are affected by any degree of anal dysplasia at the clinical, histopathological, or instrumental screening evaluation^23^.

A screening pathway should include a detailed social and medical history—especially regarding any prior anal SIL or HPV infection, a thorough inspection of the anal region, and a comprehensive digital anorectal examination (DARE)^24^. Anal swabs should be performed. Considering the resources required for HRA, its adoption currently remains limited^25^. Cytological and HPV swabs, with or without genotyping, are available; however, due to a lack of sufficient data none can currently be recommended over the others^26^. Self-testing, especially the HPV anal self-test (HPV-DNA-PCR), may be considered after appropriate patient education and it could replace most of the surveillance tests that are today necessarily provided by healthcare professionals only^27^, making access to diagnostics easier.

If pathology is detected, American guidelines recommend referral for HRA. In cases of unremarkable findings, the swab should be repeated after 12–24 months^22^. Other guidelines recommend a 12-month follow-up in patients with cytologically normal findings, but evidence of HPV^19^.

There is a growing international trend towards recommending HPV testing with genotyping as a stand-alone screening method. In regions with limited access to HRA, only those with HPV-16 positivity or ASC-US/ASC-H or HSIL findings should be referred. If HRA is not available, annual inspection and DARE may be sufficient, although this can only be considered as early detection for anal carcinoma^19,22^. To improve detection of progression to ASCC, DNA methylation analysis of anal swabs of PLWH and individuals with SILs is increasingly being undertaken and represents a promising innovation for identifying patients at particularly high risk^28^.

## HRA

HRA is the ‘gold standard’ diagnostic modality to identify and treat any HPV-induced dysplastic lesion arising in the anal canal, anal margin, or perianal zone. Through HRA, the anal canal and perianal area can be magnified with sufficient resolution to spot sub-millimetre premalignant lesions, which are reported according to a dedicated nomenclature describing the morphological appearance of HPV-induced epithelial transformations^29,30^.

**Fig. 5:**
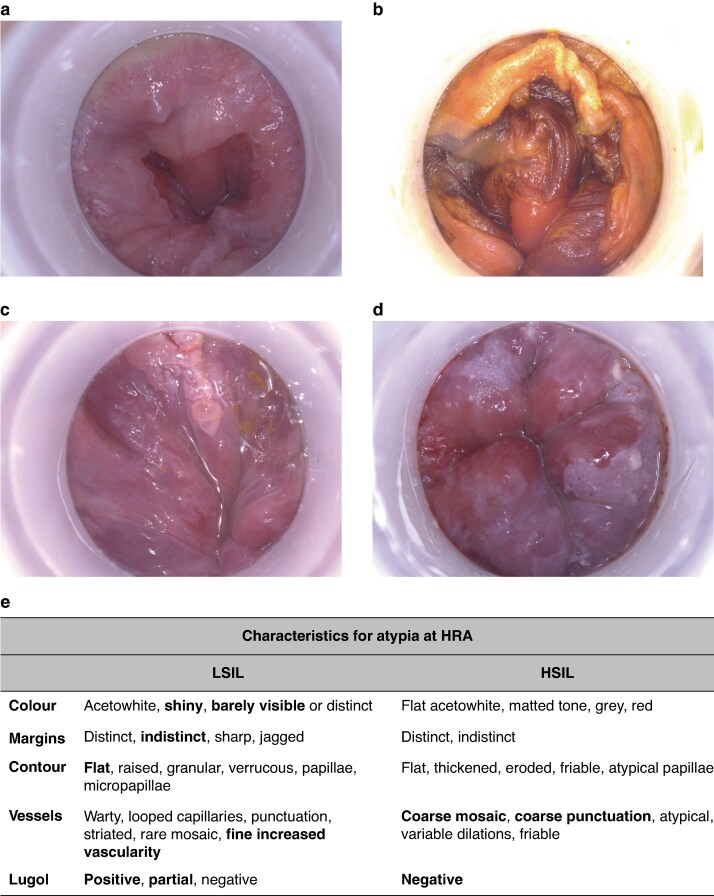
Digital HRA **a** Squamous-columnar junction after application of 5% acetic acid. **b** Squamous-columnar junction after application of Lugol solution. **c** LSIL. **d** HSIL. **e** Charactersitic of atypia using HRA: Colour describes reaction to acetic acid application, ranging from immediately visible (shiny) to difficult to identify (barely visible). Margins can be faded (indistinct) with a smooth and even pattern of growth (flat). Vascularity on the surface is usually slight (fine increased vascularity) in LSIL, while in HSIL tend to be more visible with a rough pattern centered by red spots (Coarse mosaic, coarse punctation). Lugol reaction is defined by its intensity; in HSIL Lugol normally does not stain (negative); in LSIL a dark brown reaction is seen (positive, aprtial). HRA, high-resolution anoscopy; LSIL, low-grade squamous intraepithelial lesion; HSIL, high-grade squamous intraepithelial lesion.

HRA uses a high-magnification, high-resolution colposcope or digital camera and the application of vital colours (5% acetic acid and Lugol solution) to enhance any HPV-induced lesion and to precisely visualize the squamous-columnar junction where most HPV-induced dysplastic lesions develop (*Fig. 5*). The progressive use of digital equipment has led to an expansion of diagnostic capabilities, recently integrating artificial intelligence (AI) systems that, even in their initial phase, could allow for better identification of dysplastic lesions and shorten the learning curve of HRA^31,32^.

The IANS considers that HRA with directed biopsy is the ‘gold standard’ investigation for detection of premalignant lesions, against which anal cytology and other diagnostic procedures are compared; however, high-quality evidence for the use of HRA does not exist^33^.

Magnification between ×15 and ×50 allows precise identification of dysplastic epithelium, targeted ablation, and immediate intraoperative confirmation of tissue effect. Overall, the ‘see and treat’ principle means that, if a lesion is seen, it can be treated in real time. Equally important is systematic photo and video documentation at each session, enabling objective comparison across visits. This facilitates assessment of therapeutic response, early recognition of recurrence, and optimization of subsequent treatment. It also enhances patient understanding and compliance: reviewing sequential images provides reassurance when clearance is achieved and transparency when recurrence occurs.

## HSIL treatment

Treatments for anal HPV-induced lesions should be classified according to their mechanism of action. To date, several chemical substances and ablative or excisional techniques have been adopted, alone or in combination, to clear anal dysplasia (*Table 2*).

**Table 2:** Treatment modalities for AIN

Treatment category	Technique	Mechanism of action	Indications	Advantages	Limitations and complications
Chemical (topical)	Imiquimod^34–36^	Immune response modifier (TLR-7 activation)	LSILs; selected HSILs; multifocal disease	Non-invasive; sphincter-preserving	Variable response; local irritation; recurrence
	Topical 5-fluorouracil (5-FU)^37,38^	Antimetabolite inhibiting DNA synthesis	LSILs/HSILs; extensive disease	Non-surgical option	Inflammation; patient adherence
	Trichloroacetic acid (TCA)^39^	Chemical coagulation necrosis	Small superficial lesions	Simple; inexpensive	Limited depth; multiple sessions
Ablative	Infrared coagulation (IRC)^40^	Coagulative/thermal necrosis	Discrete HSILs/LSILs	Office-based; repeatable	Recurrence; multiple treatments
	Electrocautery ablation^41,42^	Thermal destruction	HSILs; selected LSILs	Widely available	Pain; thermal spread
	CO_2_ laser ablation^43^	Tissue H_2_O vaporization	LSILs/HSILs visible on HRA	High precision	Equipment/expertise; recurrence
	Argon plasma coagulation (APC)^44^	Non-contact thermal coagulation	HSILs (HIV + MSM pilot data)	Office-based; repeatable	Pain; high recurrence; cost
Targeted phototherapy	Photodynamic therapy (PDT)^45,46^	Photosensitizer + light → selective cytotoxicity	HSILs; AIN3 (pilot/series data)	Non-invasive; tissue-selective	Limited evidence; small series
Surgical	HRA-directed local excision^47^	Complete lesion removal	Unifocal HSILs; suspected invasion	Histology; definitive removal	Scarring; Incontinence risk
	Wide local excision/mapping	Radical removal of dysplasia	Extensive or refractory HSILs	Oncological control	High morbidity; functional impact

Recurrence and metachronous disease remain frequent, irrespective of modality. HRA remains central for selection and follow-up. Ablative and targeted therapies balance efficacy and functional preservation; surgery is reserved for selected settings. AIN, anal intraepithelial neoplasia; TLR-7, toll-like receptor-7; LSILs, low-grade squamous intraepithelial lesions; HSILs, high-grade squamous intraepithelial lesions; HRA, high-resolution anoscopy; HIV, human immunodeficiency virus; MSM, men who have sex with men.

LSILs rarely represent a major therapeutic challenge. In immunocompetent and younger patients infected with low-risk HPV subtypes, spontaneous regression is common, making expectant management both safe and reasonable. Topical therapies such as imiquimod, 5-fluorouracil, and podophyllotoxin have been investigated, but efficacy remains modest and recurrence is frequent. In many countries, these agents are not licensed for intra-anal use, restricting their application^48,49^. When topical therapy is chosen, its effectiveness depends on patient adherence, tolerability, and structured follow-up to assess true benefit. Compliance is often a limiting factor and adverse local reactions can reduce tolerability, meaning that topical therapy is rarely sufficient as a stand-alone approach in patients with HSILs.

Ablative therapy has therefore become the mainstay of HSIL management. Cryotherapy induces intracellular ice formation, vascular injury, and local inflammatory repair, and, although clearance can be achieved in 50–60% of lesions, recurrence rates approach 50% at 5 years^50^. In MSM and transplant recipients, clearance rates are lower and repeated interventions are frequently required. Argon plasma coagulation and radio-frequency ablation are effective alternatives, achieving clearance rates of 60–80%, but require specialist facilities and anaesthesia, and incur considerable cost, restricting their use to referral centres^51,52^.

CO_2_ laser ablation has emerged as an attractive option due to precise energy delivery, limited lateral heat diffusion, and preservation of normal tissue. The laser beam, absorbed by intracellular water, produces controlled electrovaporization and carbonization with minimal fibrosis and mucosal scarring. Several clinical series have reported short-term remission rates above 80%, although recurrence remains 20–40% at 1 year, reflecting the recurrent and multifocal nature of HPV disease, rather than technical failure^53–55^. In a prospective trial of 52 HIV-positive patients with previously untreated HSILs, a single CO_2_ laser session achieved a complete response in 50% and a partial response in 20.8%, with older age and low CD4 counts predicting poorer outcomes^55^. Larger retrospective cohorts have confirmed that repeated laser sessions are often necessary, but well tolerated, with acceptable morbidity and durable functional outcomes. These findings suggest that laser therapy, although not curative in the strictest sense, represents a reproducible and safe strategy for long-term disease control.

Surgical excision remains an option for well-circumscribed macroscopic lesions, but recurrence occurs in 30–50% within 2 years and repeated procedures risk sphincter injury^56^. For multifocal disease, excision is rarely appropriate, as wide resection carries a high risk of morbidity and functional compromise.

Despite the lower morbidity and greater acceptability of topical and ablative treatments, it is important to consider that surgical excision alone allows for regular monitoring of the underlying pathology, providing confirmation that is not otherwise available when other treatment strategies are implemented. For this reason, if invasive or microinvasive disease is ever suspected, ablative or topical treatments should be avoided.

Recurrence after treatment remains a major challenge. Immunosuppressed populations, particularly HIV-positive MSM and transplant recipients, experience the highest number of recurrences and often require repeat therapy every 6–12 months. By contrast, immunocompetent individuals generally require fewer procedures. Elderly women, who could benefit most from minimally invasive therapy, are seldom screened and typically present late with overt lesions visible without magnification, which are too often managed with surgical excision^57^.

Overall, anal dysplasia should be regarded as a chronic, multifocal disease. No single modality ensures durable control and optimal management requires a multimodal approach: targeted HRA-guided ablation, selective topical therapy, structured image-based surveillance, and preventive vaccination. HRA with systematic imaging offers opportunities to improve oncological safety, but also establish a reproducible framework for long-term management, balancing efficacy with functional preservation and patient engagement. This philosophy transforms anal dysplasia care from isolated therapeutic activities into an integrated, chronic disease management programme, where vigilance, precision, and persistence are essential to achieving sustained control.

The incidence of ASCC is increasing, with extremely high peaks in immunosuppressed patients. As for cervical cancer, HPV vaccination campaigns will reduce the incidence of anal cancer, although several decades will be needed to achieve significant results. In the meantime, it is essential to implement screening programmes for those identified as high risk. Increasing knowledge with regard to the natural history and histopathological characteristics of this disease is making the treatment of premalignant lesions progressively more precise. HRA represents the most important diagnostic tool in the screening, identification, and treatment of HSILs; the development of digital technology and AI systems could increase its widespread adoption and ease of use, potentially reducing the progression of HSILs to invasive cancer.

## Data Availability

Not applicable.

## References

[1] Gondal TA, Chaudhary N, Bajwa H, Rauf A, Le D, Ahmed S. Anal cancer: the past, present and future. Curr Oncol 2023;30:3232–3250 36975459 10.3390/curroncol30030246 PMC10047250

[2] National Cancer Institute: Surveillance, Epidemiology, and End Results Program . *Cancer Stat Facts: Anal Cancer*. https://seer.cancer.gov/statfacts/html/anus.html (accessed 10 March 2023)

[3] Loopik DL, Bentley HA, Eijgenraam MN, IntHout J, Bekkers RLM, Bentley JR. The natural history of cervical intraepithelial neoplasia grades 1, 2, and 3: a systematic review and meta-analysis. J Low Genit Tract Dis 2021;25:221–231 34176914 10.1097/LGT.0000000000000604

[4] Arbyn M, Xu L, Simoens C, Martin-Hirsch PP. Prophylactic vaccination against human papillomaviruses to prevent cervical cancer and its precursors. Cochrane Database Syst Rev 2018; (5) CD009069 29740819 10.1002/14651858.CD009069.pub3 PMC6494566

[5] Kamolratanakul S, Pitisuttithum P. Human papillomavirus vaccine efficacy and effectiveness against cancer. Vaccines (Basel) 2021;9:1413 34960159 10.3390/vaccines9121413 PMC8706722

[6] Darragh TM, Colgan TJ, Cox JT, Heller DS, Henry MR, Luff RD et al The Lower Anogenital Squamous Terminology Standardization Project for HPV-associated lesions: background and consensus recommendations from the College of American Pathologists and the American Society for Colposcopy and Cervical Pathology. J Low Genit Tract Dis 2012;16:205–242 [Erratum in: J Low Genit Tract Dis 2013;17:368] 22820980 10.1097/LGT.0b013e31825c31dd

[7] Bean SM, Chhieng DC. Anal-rectal cytology: a review. Diagn Cytopathol 2010;38:538–546 19941374 10.1002/dc.21242

[8] Chin-Hong PV, Berry JM, Cheng SC, Catania JA, Da Costa M, Darragh TM et al Comparison of patient- and clinician-collected anal cytology samples to screen for human papillomavirus-associated anal intraepithelial neoplasia in men who have sex with men. Ann Intern Med 2008;149:300–306 18765699 10.7326/0003-4819-149-5-200809020-00004

[9] Salit IE, Lytwyn A, Raboud J, Sano M, Chong S, Diong C et al The role of cytology (Pap tests) and human papillomavirus testing in anal cancer screening. AIDS 2010;24:1307–1313 20442633 10.1097/QAD.0b013e328339e592

[10] Clarke MA, Deshmukh AA, Suk R, Roberts J, Gilson R, Jay N et al A systematic review and meta-analysis of cytology and HPV-related biomarkers for anal cancer screening among different risk groups. Int J Cancer 2022;151:1889–1901 [Erratum in: Int J Cancer 2023;153:E2] 35793241 10.1002/ijc.34199 PMC9588562

[11] Albuquerque A, Rios E, Macedo G. The impact of P16 immunostaining in reducing anal squamous intraepithelial lesions indication for treatment. Am J Surg Pathol 2017;41:1151–1152 28505001 10.1097/PAS.0000000000000858

[12] Liu Y, McCluggage WG, Darragh TM, Farhat N, Blakely M, Sigel K et al p16 immunoreactivity correlates with morphologic diagnosis of HPV-associated anal intraepithelial neoplasia: a study of 1000 biopsies. Am J Surg Pathol 2021;45:1573–1578 34231547 10.1097/PAS.0000000000001769

[13] Jongen VW, Richel O, Marra E, Siegenbeek van Heukelom ML, van Eeden A, de Vries HJC et al Anal squamous intraepithelial lesions (SILs) in human immunodeficiency virus-positive men who have sex with men: incidence and risk factors of SIL and of progression and clearance of low-grade SILs. J Infect Dis 2020;222:62–73 31755920 10.1093/infdis/jiz614

[14] English NC, Warden C. Epidemiology of anal cancer. Surg Oncol Clin N Am 2025;34:11–19 39547763 10.1016/j.soc.2024.07.011

[15] Szymonowicz KA, Chen J. Biological and clinical aspects of HPV-related cancers. Cancer Biol Med 2020;17:864–878 33299640 10.20892/j.issn.2095-3941.2020.0370 PMC7721094

[16] McCredie MR, Sharples KJ, Paul C, Baranyai J, Medley G, Jones RW et al Natural history of cervical neoplasia and risk of invasive cancer in women with cervical intraepithelial neoplasia 3: a retrospective cohort study. Lancet Oncol 2008;9:425–434 18407790 10.1016/S1470-2045(08)70103-7

[17] Stoler MH . Human papillomaviruses and cervical neoplasia: a model for carcinogenesis. Int J Gynecol Pathol 2000;19:16–28 10638450 10.1097/00004347-200001000-00004

[18] Doorbar J . Papillomavirus life cycle organization and biomarker selection. Dis Markers 2007;23:297–313 17627064 10.1155/2007/613150 PMC3851388

[19] Chromy D, Aigner F, Becker JC, Bickel M, Brunner A, Classen J et al German-Austrian guideline on screening for anal dysplasia and anal carcinoma in people living with HIV. J Dtsch Dermatol Ges 2025;23:1025–1040 40320909 10.1111/ddg.15719 PMC12338428

[20] Palefsky JM, Lee JY, Jay N, Goldstone SE, Darragh TM, Dunlevy HA et al Treatment of anal high-grade squamous intraepithelial lesions to prevent anal cancer. N Engl J Med 2022;386:2273–2282 35704479 10.1056/NEJMoa2201048 PMC9717677

[21] Wei F, Gaisa MM, D'Souza G, Xia N, Giuliano AR, Hawes SE et al Epidemiology of anal human papillomavirus infection and high-grade squamous intraepithelial lesions in 29 900 men according to HIV status, sexuality, and age: a collaborative pooled analysis of 64 studies. Lancet HIV 2021;8:e531–e543 34339628 10.1016/S2352-3018(21)00108-9 PMC8408042

[22] Stier EA, Clarke MA, Deshmukh AA, Wentzensen N, Liu Y, Poynten IM et al International Anal Neoplasia Society’s consensus guidelines for anal cancer screening. Int J Cancer 2024;154:1694–1702 38297406 10.1002/ijc.34850

[23] Binda GA, Gagliardi G, Dal Conte I, Verra M, Cassoni P, Cavazzoni E et al Practice parameters for the diagnosis and treatment of anal intraepithelial neoplasia (AIN) on behalf of the Italian Society of Colorectal Surgery (SICCR). Tech Coloproctol 2019;23:513–528 31243606 10.1007/s10151-019-02019-5

[24] Hillman RJ, Berry-Lawhorn JM, Ong JJ, Cuming T, Nathan M, Goldstone S et al International Anal Neoplasia Society guidelines for the practice of digital anal rectal examination. J Low Genit Tract Dis 2019;23:138–146 30907777 10.1097/LGT.0000000000000458

[25] Benevolo M, Giuliani M, Giorgi Rossi P, Rollo F, Giuliani E, Stingone C et al High-resolution anoscopy referral rates adopting different anal cancer screening strategies for men who have sex with men. Cancer Prev Res 2025;18:291–298 10.1158/1940-6207.CAPR-24-0435 39936264

[26] Compton ML . “Other” high-risk HPV: challenges in anal cancer screening. Cancer Cytopathol 2025;133:e70025 10.1002/cncy.70025 40439347

[27] Biasioli L, Rossotti R, Tavelli A, De Bona A, Tincati C, Calzavara D et al Performance evaluation of a self-administered point-of-care test for anal HPV screening in PrEP users: data from a community-based PrEP service. Sex Transm Infect 2024;100:252–255 38641362 10.1136/sextrans-2023-055939

[28] Dias Gonçalves Lima F, Rozemeijer K, van der Zee RP, Dick S, Ter Braak TJ, Geijsen DE et al DNA methylation analysis on anal swabs for anal cancer screening in people living with human immunodeficiency virus. J Infect Dis 2025; 232:1040–1049 39718982 10.1093/infdis/jiae627

[29] Hillman RJ, Cuming T, Darragh T, Nathan M, Berry-Lawthorn M, Goldstone S et al 2016 IANS international guidelines for practice standards in the detection of anal cancer precursors. J Low Genit Tract Dis 2016;20:283–291 27561134 10.1097/LGT.0000000000000256

[30] Camus M, Lesage AC, Fléjou JF, Hoyeau N, Atienza P, Etienney I. Which lesions should be biopsied during high-resolution anoscopy? Prospective descriptive study of simple morphological criteria. J Low Genit Tract Dis 2015;19:156–160 24983348 10.1097/LGT.0000000000000064

[31] Santorelli C, Leo CA, Hodgkinson JD, Baldelli F, Cantarella F, Cavazzoni E. Screening for squamous cell anal cancer in HIV positive patients: a five-year experience. J Invest Surg 2018;31:378–384 28644711 10.1080/08941939.2017.1334845

[32] Saraiva MM, Spindler L, Fathallah N, Beaussier H, Mamma C, Quesnée M et al Artificial intelligence and high-resolution anoscopy: automatic identification of anal squamous cell carcinoma precursors using a convolutional neural network. Tech Coloproctol 2022;26:893–900 35986806 10.1007/s10151-022-02684-z

[33] Cho SD, Groves E, Lao VV. History of high-resolution anoscopy. Clin Colon Rectal Surg 2018;31:336–346 30450017 10.1055/s-0038-1668103 PMC6237179

[34] Wieland U, Brockmeyer NH, Weissenborn SJ, Hochdorfer B, Stücker M, Swoboda J et al Imiquimod treatment of anal intraepithelial neoplasia in HIV-positive men. J Acquir Immune Defic Syndr 2006;42:180–185 10.1001/archderm.142.11.1438 17116834

[35] Richel O, de Vries HJC, van Noesel CJM, Dijkgraaf MGW, Prins JM. Comparison of imiquimod, topical fluorouracil, and electrocautery for AIN in HIV-positive MSM: open-label RCT. Lancet Oncol 2013;14:346–353 23499546 10.1016/S1470-2045(13)70067-6

[36] Fox PA, Nathan M, Francis N, Singh N, Weir J, Dixon G et al Imiquimod for high-grade anal intraepithelial neoplasia in HIV + MSM on HAART. AIDS 2010;24:1257–1265 10.1097/QAD.0b013e32833d466c 20729710

[37] Richel O, Wieland U, de Vries HJ, Brockmeyer NH, van Noesel C, Potthoff A et al Topical 5-fluorouracil for AIN in HIV-positive men. Br J Dermatol 2010;162:393–397 10.1111/j.1365-2133.2010.09982.x 20716208

[38] Snyder SM, Siekas L, Aboulafia DM. Initial Experience with Topical Fluorouracil for Treatment of HIV-Associated Anal Intraepithelial Neoplasia. J Int Assoc Physicians AIDS Care (Chic) 2011;10:83–88 21266323 10.1177/1545109710382578

[39] Weis SE . Current treatment options for anal intraepithelial neoplasia. Onco Targets Ther. 2013;10:651–665 10.2147/OTT.S38217 PMC3684220 23788834

[40] Goldstone SE, Lensing SY, Stier EA, Darragh T, Lee JY, van Zante A et al Randomised clinical trial of infrared coagulation ablation vs monitoring for HSIL in HIV adults. Clin Infect Dis 2019;68:1204–1212 30060087 10.1093/cid/ciy615 PMC6588032

[41] Burgos J, Curran A, Landolfi S, Navarro J, Tallada N, Guelar A et al Electrocautery ablation of HSIL in HIV + MSM. HIV Med 2016;17:524–531 26688291 10.1111/hiv.12352

[42] Sigel KM . Electrocautery ablation of anal HSIL: outcomes and experience. HIV Clin Trials 2020;21:121–129

[43] Watemberg S, Landau O, Avrahami R, Kaplan I, Giler S, Kott I. Successful treatment of anal tumors with CO2 laser in elderly, high-risk patients. J Clin Laser Med Surg 1996;14:115–117 9484086 10.1089/clm.1996.14.115

[44] de Pokomandy A, Rouleau D, Lalonde R, Beauvais C, de Castro C, Coutlée F et al Argon plasma coagulation treatment of anal high-grade squamous intraepithelial lesions in men who have sex with men living with HIV: results of a 2-year prospective pilot study. HIV Med. 2018;19:81–89 28833949 10.1111/hiv.12544

[45] Welbourn H, Duthie G, Powell J, Moghissi K et al Can photodynamic therapy be the preferred treatment option for anal intraepithelial neoplasia? Initial results of a pilot study. Photodiagnosis Photodyn Ther 2014;11:20–21 24280437 10.1016/j.pdpdt.2013.11.004

[46] Hamdan KA, Tait IS, Nadeau V, Padgett L, Carey F, Steele RJ. Treatment of grade III AIN with photodynamic therapy: case report. Dis Colon Rectum 2003;46:1555–1559 14605579 10.1007/s10350-004-6813-9

[47] Chang GJ, Berry JM, Jay N, Palefsky JM, Welton ML. Surgical treatment of high-grade anal squamous intraepithelial lesions: a prospective study. Dis Colon Rectum 2002;45:453–458 12006924 10.1007/s10350-004-6219-8

[48] Fox PA, Nathan M, Francis N, Singh N, Weir J, Dixon G et al A double-blind, randomized controlled trial of the use of imiquimod cream for the treatment of anal canal high-grade anal intraepithelial neoplasia in HIV-positive MSM on HAART, with long-term follow-up data including the use of open-label imiquimod. AIDS 2010;24:2331–2335 20729710 10.1097/QAD.0b013e32833d466c

[49] Fuertes I, Bastida C, Lopez-Cabezas C, Rodríguez-Carunchio L, Ordi J, Mallolas J et al The effectiveness and tolerability of imiquimod suppositories to treat extensive intra-anal high-grade squamous intraepithelial lesions/warts in HIV-infected individuals. Int J STD AIDS 2019;30:1194–1200 31558130 10.1177/0956462419864506

[50] Braga EA, Filho LG, Saad SS. Argon plasma versus electrofulguration in the treatment of anal and perianal condylomata acuminata in patients with acquired immunodeficiency virus. Acta Cir Bras 2017;32:482–490 [Erratum in: Acta Cir Bras 2017;28:0] 28700010 10.1590/s0102-865020170060000009

[51] Gao Y, Chu W, Hou L, Cheng J, Zhong G, Xia B et al Comparing the effects of argon plasma coagulation and interferon therapy in patients with vaginal intraepithelial neoplasia: a single-center retrospective study. Arch Gynecol Obstet 2024;310:561–569 38683394 10.1007/s00404-024-07477-3 PMC11168973

[52] Albuquerque A . Argon plasma coagulation as treatment for anal condylomas: a narrative review. J Low Genit Tract Dis 2024;28:198–201 38518218 10.1097/LGT.0000000000000805

[53] Goldstone SE, Lensing SY, Stier EA, Darragh T, Lee JY, van Zante A et al A randomized clinical trial of infrared coagulation ablation versus active monitoring of intra-anal high-grade dysplasia in adults with human immunodeficiency virus infection: an AIDS malignancy consortium trial. Clin Infect Dis 2019;68:1204–1212 30060087 10.1093/cid/ciy615 PMC6588032

[54] van Heukelom ML S, Gosens KCM, Prins JM, de Vries HJC. Cryotherapy for intra- and perianal high-grade squamous intraepithelial lesions in HIV-positive men who have sex with men. Am J Clin Dermatol 2018;19:127–132 28695429 10.1007/s40257-017-0311-z PMC5797558

[55] Fuertes I, Chivite I, Cranston RD, Sánchez E, Cordón E, Rodríguez-Carunchio L et al Short-term effectiveness and tolerability of carbon dioxide laser for anal high-grade squamous intraepithelial lesions in individuals living with HIV. Int J STD AIDS 2022;33:709–717 35611790 10.1177/09564624221100069

[56] Cajas-Monson LC, Ramamoorthy SL, Cosman BC. Expectant management of high-grade anal dysplasia in people with HIV: long-term data. Dis Colon Rectum 2018;61:1357–1363 30346366 10.1097/DCR.0000000000001180

[57] Brogden DRL, Walsh U, Pellino G, Kontovounisios C, Tekkis P, Mills SC. Evaluating the efficacy of treatment options for anal intraepithelial neoplasia: a systematic review. Int J Colorectal Dis 2021;36:213–226 32979069 10.1007/s00384-020-03740-6 PMC7801290

